# Supplementary material for: Engineering an autonomous VH domain to modulate intracellular pathways and to interrogate the eIF4F complex
Source: Nat Commun. 2022 Aug 18;13:4854. doi: 10.1038/s41467-022-32463-1 (PMC9388512; doi:10.1038/s41467-022-32463-1)
Supplement: Supplementary file 1 — Supplementary Information [file 41467_2022_32463_MOESM1_ESM.pdf]

Name	Sequence
4D5 VH	MKKNIAFLLASMFVFSIATNAYASEVQLVESGGGLVQPGGSLRLSCAASGFNIKDTYIHWVRQAPGKGLEWVARIYPTNGYTRYADSVKGRFTISADTSKNTAYLQMNSLRAEDTAVYYCSRWGGDGFYAMDYWGQGTLVTVSSAHHHHHH*
VH-33	MKKNIAFLLASMFVFSIATNAYASEVQLVESGGGLVQPGGSLRLSCAASGFNIKDTYIHWVRQAPGKGLEWVASIYPTNGYTRYADSVKGRFTISADTSKNTVYLQMNSLRAEDTAVYYCGRWGGDGFYPMDYWGQGTLVTVSSAHHHHHH*
VH-36	MKKNIAFLLASMFVFSIATNAYASEVQLVESGGGLVQPGGSLRLSCAASGFNIKDTYIDWVRQAPGKGLEWVARIYPTNGYTRYADSVKGRFTISADTSKNTAYLQMNSLRAEDTAVYYCGRWGGDGFYPMDYRGQGTLVTVSSAHHHHHH*
VH-36i	MSEVQLVESGGGLVQPGGSLRLSCAASGFNIKDTYIDWVRQAPGKGLEWVARIYPTNGYTRYADSVKGRFTISADTSKNTAYLQMNSLRAEDTAVYYCGRWGGDGFYPMDYRGQGTLVTVSSAHHHHHH*
VH-36i.1	MSEVQLVESGGGLVQPGGSLRLSSACSGFNIKDTYIDWVRQAPGKGLEWVARIYPTNGYTRYADSVKGRFTISADTSKNTAYLQMNSLRAEDTAVYYTGRWGGDGFYPMDYRGQGTLVTVSSGGAHHHHHH*
VH-37i	MSEVQLVESGGGLVQPGGSLRLSSACSGFNIKDTYIDWVRQAPGKGLEWVARIYPTNGYTRYADSVKGRFTISADTSKNTAYLQMNSLRAEDTAVYYTGRSSSAMDYRGQGTLVTVSSGGAHHHHHH*
VH-37i.1	MSEVQLVESGGGLVQPGGSLRLSSACSGFNIKDTYIDWVRQAPGKGLEWVARIYPTNGYTRYADSVKGRFTISADTSKNTVYLQMNSLRAEDTAVYYTGRSSSAMDYRGQGTLVTVSSGGAHHHHHH*
VH-37i.2	MSEVQLVESGGGLVQPGGSLRLSSACSGFNIKDTYIDWVRQAPGKGLEWVARIYPTNGYTRYADSVKGRFTISADTSKNTAYLQMNSLRAEDTAVYYTVRSSSAMDYRGQGTLVTVSSGGAHHHHHH*
VH-38i	MSEVQLVESGGGLVQPGGSLRLSSACSGFNIKDTYIDWVRQAPGKGLEWVARIYPTNGYTRYADSVKGRFTISADTSKNTVYLQMNSLRAEDTAVYYTVRSSSAMDYRGQGTLVTVSSGGAHHHHHH*
VH-38i.1	MSEVQLVESGGGLVQPGGSLRLSSAISGFNIKDTYIDWVRQAPGKGLEWVARIYPTNGYTRYADSVKGRFTISADTSKNTVYLQMNSLRAEDTAVYYTVRSSSAMDYRGQGTLVTVSSGGAHHHHHH*

Supplementary Table 1: Sequences of the VH domains used in this study. The N-terminal signal peptide and the C-terminal His-tag are underlined.

Name	CDR1	CDR2	CDR3	Number of clones	K_d (µM)
1A2	SISSTS	SPSSGSTS	GRMAYSYPSSFSASSFDLYYAFD	2	**0.044**
1A7	SFYYTY	YPSNGYTY	GRYYSSAFFYSPTNSYSAFD	3	**N.T.**
1A8	TFKYTY	FPVLGYTY	GRFSASSSYAFD	1	**0.244**
1A12	FIVSTP	YPFNGSTK	VRSFASYFNPLSVSYSYLVAHAVD	1	**7.1**
1C5	SISSTS	SPSSGSTS	GRVAKDLNSSSPSFVVNTYSSFGFD	7	**0.078**
1C11	YFPHTA	YPYYGTTF	GRYPYTFFVGMD	1	**0.213**
1E7	DVYDTA	FPVIGYTS	VRLAYSADFAASEVSSAID	1	**3.67**
2A3	SIDATY	YPASGITA	GRFFSNPDFAVD	1	**0.360**
2D4	VVYQTY	YPASGYTY	GRYYASPLYFQNFFVFFSNGID	1	**15.4**

Supplementary Table 2: VH domain phage selected clones. List of eIF4E interacting VH domains identified from phage panning experiments against the naïve VH domain library. Selected CDR1,2 and 3 sequences and clone frequency denoted. VH domains were cloned by PCR into peT22B vectors for bacteria protein expression and purification and screened using bilayer interferometry (ForteBio Blitz). Interferometry data was used to determine preliminary dissociation constants (K_d). NT = not tested.

	8 mins	60 mins	110 mins	M1	S2	V7	L12	G17	S24	S35	S73	K78	N79	Q84	M85	N86	T94	Y97	R100	S108	N115	S118	F120	Y124
**VH-M4^{F120L}**	10	0	0																				**L**	
**VH-M4^{F120V}**	5	0	0																				**V**	
**VH-M4^{Y97S}**	5	0	0															**S**						
**VH-M4$^{Y97C/F120L}$**	4	14	4															**C**					**L**	
**VH-M4^{F120I}**	4	4	0																				**I**	
**VH-M4$^{G17N/F120I}$**	2	0	0					**N**															**I**	
**VH-M4$^{S24G/F120I}$**	2	4	1						**G**														**I**	
**VH-M4$^{T94A/F120I}$**	1	0	0														**A**						**I**	
**VH-M4$^{S2N/V7G/L12R/F120V}$**	1	0	0		**N**	**G**	**R**																**I**	
**VH-M4$^{S73G/Y97S}$**	1	0	0								**G**							**S**						
**VH-M4$^{G17D/F120L}$**	1	0	0					**D**															**L**	
**VH-M4$^{Q84R/M85V/S118G/F120I}$**	1	0	0											**R**	**V**							**G**	**I**	
**VH-M4$^{R100H/F120I}$**	1	1	0																**H**				**I**	
**VH-M4$^{120L/Y124C}$**	1	0	0																				**L**	**C**
**VH-M4$^{M1I/N115S/F120I}$**	1	0	0	**I**																	**S**		**I**	
**VH-M4$^{N79S/F120L}$**	1	0	0										**S**										**L**	
**VH-M4$^{S24G/F120L}$**	1	0	0						**G**														**L**	
**VH-M4$^{K78R/F120L}$**	1	0	0									**R**											**L**	
**VH-M4$^{S108R/F120I}$**	1	4	34																	**R**			**I**	
**VH-M4$^{S35C/F120L}$**	1	0	0							**C**													**L**	
**VH-M4^{N86P}**	1	0	0													**P**								
**VH-M4$^{S108R/F120L}$**	0	1	0																	R			**L**	
**Frameshift**	0	0	9																					
**Sequenced Clones**	46	28	48																					

Supplementary Table 3: The table shows the analysis of the sequencing data from each round of selection with associated mutations. The number of unique clones identified in the table are given for each maturation selection round on the left-hand side of the table with total number of sequenced cloned denoted in the last row. Approximately 30-50 yeast isolates were sequenced each round. The most enriched sequence from the last round is highlighted in red (VH-M4$^{S108R/F120I}$ also termed VH-S4 in manuscript**)**. The right-hand side of the table indicates the mutations introduced by the random error mutagenesis and selected for by the rounds of maturation selection.

PDB ID:	7D8B	7D6Y	7XTP
Resolution (Å)	70.2- 2.46, (2.59-2.46)	44.22-1.67, 9, (1.76-1.67)	46.29-1.85 (1.89-1.85)
Space Group	$P2_1$	C2	$P2_1$
Unit Cell Dimensions (Å)	a = 47.60, b = 140.39, c = 61.96, α = γ = 90°, β = 140.39	a = 198.85, b = 45.32, c = 39.76, α = γ = 90°, β = 93.19	a = 75.62, b = 57.21, c = 78.96, α = γ = 90°, β = 93.94
Temp (K)	100	100	100
Redundancy	7.7, (7.6)	3.7. (3.7)	7.0 (6.9)
Unique Collected Reflections	28611, (4143)	41261, (5689)	59284, 3593
Completeness (%)	99.9, (99.9)	99.4, (96.2)	99.2, (97.5)
R Sym (%)	0.177, (0.876)	0.116, (0.673)	0.147, (1.691)
I/sigma	3.6, (0.9)	6.1, (1.1)	11.8, (1.3)
R factor (%)	25.00	18.15	22.65
R free (%)	29.18	20.64	25.99
RMS Bonds (Å)	0.0022	0.0039	0.0023
RMS Angles (°)	1.185	1.197	1.171
Wilson B-factor ($Å^2$)	31.06	3.26	18.7
Average Refined B Factors			
Chain A	40.88, (eIF4E)	13.14, (eIF4E)	27.28, (eIF4E)
Chain B	34.44, (VH-S4)	10.22, (VH-1C5)	20.79, (VH-S4ss)
Chain C	38.59, (eIF4E)	-	25.62, (eIF4E)
Chain D	33.57, (VH-S4)	-	19.68, (VH-S4ss)
		18.41, (m^7GTP)	27.5, (m^7GTP)
		21.98, (MES)	30.08, (m^7GTP)
Waters	29.64	22.87	26.72
Number of Water Molecules	102	408	388
Ramachandran Data (Rampage). Number of Residues in (%):			
Favoured Region	99.0	98.4	98.0
Allowed Region	1.0	1.6	2.0
Outlier Region	0	0	0

Supplementary Table 4: Crystallographic data collection and refinement statistics. Highest resolution bin data stated in parentheses. Validation files provided in Supplementary Data files

Name	Sequence
VH-1C5	atgagcgaagtgcagctggtggaaagcggcggcggcctggtgcagccgggcggcagcctgcgcctgagcagcgcgattagcggctttagcattagcagcaccagcattgattgggtgcgccaggcgccgggcaaaggcctggaatgggtggcgcgcattagcccgagcagcggcagcaccagctatgcggatagcgtgaaaggccgctttaccattagcgcggataccagcaaaaacaccgtgtatctgcagatgaacagcctgcgcgcggaagataccgcggtgtattataccggccgcgtggcgaaagatctgaacagcagcagcccgagctttgtggtgaacacctatagcagctttggctttgattatcgcggccagggcaccctggtgaccgtgagcagcggcgcggcg
VH-1A2	atgagcgaagtgcagctggtggaaagcggcggcggcctggtgcagccgggcggcagcctgcgcctgagcagcgcgattagcggctttagcattagcagcaccagcattgattgggtgcgccaggcgccgggcaaaggcctggaatgggtggcgcgcattagcccgagcagcggcagcaccagctatgcggatagcgtgaaaggccgctttaccattagcgcggataccagcaaaaacaccgtgtatctgcagatgaacagcctgcgcgcggaagataccgcggtgtattataccggccgcatggcgtatagctatccgagcagctttagcgcgagcagctttgatctgtattatgcgtttgattatcgcggccagggcaccctggtgaccgtgagcagcggcgcggcg
VH-M4	atgagcgaagtgcagctggtggaaagcggcggcggcctggtgcagccgggcggcagcctgcgcctgagcagcgcgattagcggctttagcattagcagcaccagcattgattgggtgcgccaggcgccgggcaaaggcctggaatgggtggcgcgcattagcccgagcagcggcagcaccagctatgcggatagcgtgaaaggccgctttaccattagcgcggataccagcaaaaacaccgtgtatctgcagatgaacagcctgcgcgcggaagataccgcggtgtattataccggccgcgtggcgaaagctctgaacagcagcagcccgagctttgtggtgaacacctatagcagctttggctttgattatcgcggccagggcaccctggtgaccgtgagcagcggcgcggcg
VH-S2	atgagcgaagtgcagctggtggaaagcggcggcggcctggtgcagccgggcggcagcctgcgcctgagcagcgcgattagcggctttagcattagcagcaccagcattgattgggtgcgccaggcgccgggcaaaggcctggaatgggtggcgcgcattagcccgagcagcggcagcaccagctatgcggatagcgtgaaaggccgctttaccattagcgcggataccagcaaaaacaccgtgtatctgcagatgaacagcctgcgcgcggaagataccgcggtgtattataccggccgcgtggcgaaagctctgaacagcagcagcccgagctttgtggtgaacacctatagcagctttatttttgattatcgcggccagggcaccctggtgaccgtgagcagcggcgcggcg
VH-S4	atgagcgaagtgcagctggtggaaagcggcggcggcctggtgcagccgggcggcagcctgcgcctgagcagcgcgattagcggctttagcattagcagcaccagcattgattgggtgcgccaggcgccgggcaaaggcctggaatgggtggcgcgcattagcccgagcagcggcagcaccagctatgcggatagcgtgaaaggccgctttaccattagcgcggataccagcaaaaacaccgtgtatctgcagatgaacagcctgcgcgcggaagataccgcggtgtattataccggccgcgtggcgaaagccctgaacagccgcagcccgagctttgtggtgaacacctatagcagcattggctttgattatcgcggccagggcaccctggtgaccgtgagcagcggcgcggcg
VVHS4ss	atgagcgaagtgcagctggtggaaagcggcggcggcctggtgcagccgggcggcagcctgcgcctgagctgcgcgattagcggctttagcattagcagcaccagcattgattgggtgcgccaggcgccgggcaaaggcctggaatgggtggcgcgcattagcccgagcagcggcagcaccagctatgcggatagcgtgaaaggccgctttaccattagcgcggataccagcaaaaacaccgtgtatctgcagatgaacagcctgcgcgcggaagataccgcggtgtattattgcggccgcgtggcgaaagccctgaacagccgcagcccgagctttgtggtgaacacctatagcagcattggctttgattatcgcggccagggcaccctggtgaccgtgagcagcggcgcggcg
VH-1C5S^{cr}	atgagcgaagtgcagctggtggaaagcggcggcggcctggtgcagccgggcggcagcctgcgcctgagcagcgcgattagcggctttagcattagcagcaccagcattgattgggtgcgccaggcgccgggcaaaggcctggaatgggtggcgcgcattagcccgagcagcggcagcaccagctatgcggatagcgtgaaaggccgctttaccattagcgcggataccagcaaaaacaccgtgtatctgcagatgaacagcctgcgcgcggaagataccgcggtgtattataccggccgcggcagcgtgctgagcgtggcgtttaaaccgagcgtggatagctttagcagcttttataacaccaacgattatcgcggccagggcaccctggtgaccgtgagcagcggcgcggcg

Supplementary Table 5: eIF4E VH Domain interacting sequences. Nucleotide sequences used for the described eIF4E interacting and control VH domains.

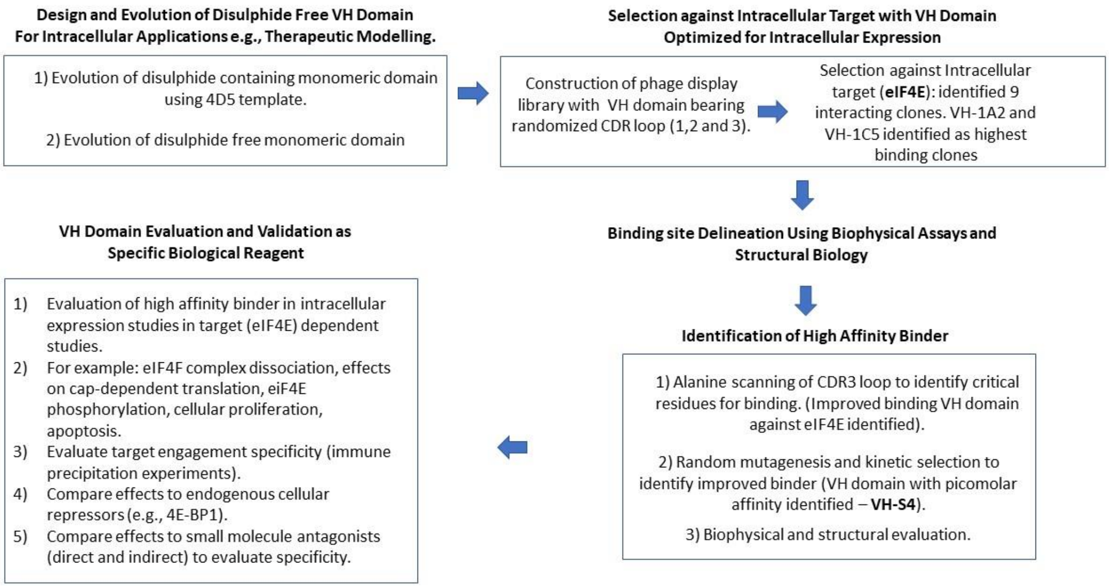

Supplementary Figure 1: VH Domain development, hit identification, evaluation and validation work flow. Outline of experimental pipeline beginning with design of an optimized VH framework, to generation of phage library, followed by selection against an intracellular target of therpuetic interest (eIF4E) , to development of a high affinity VH domain binder and completed with VH evaluation and validation experiments relevevant to the selected target.

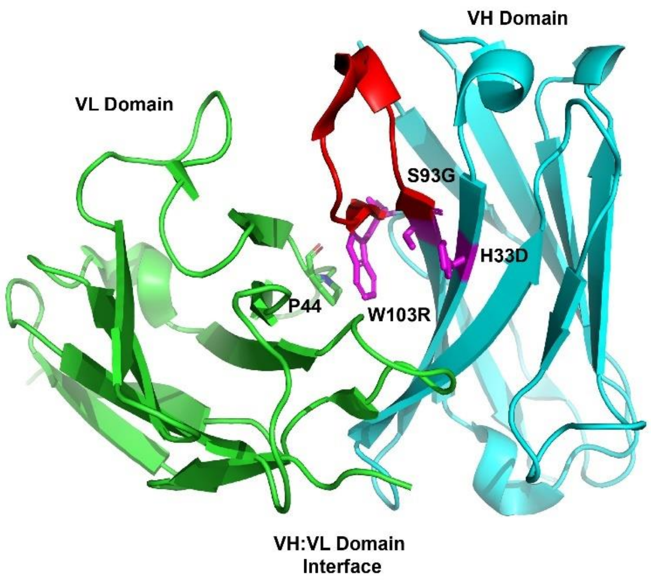

Supplementary Figure 2: Location of the mutations found in VH-36i at the former VH and VL domain interface. Structure of VH domain in complex with the VL domain, extracted from the FAB fragment crystal structure of Trastuzumab (PDB code: 6BAE), highlighting the location of the mutations found in VH-36i on the former interface with V_L (shown in stick representation, highlighted in magenta). Both H33D and W103R are located at the former VH and VL domain interface and mainly improve thermal stability of the VH variant by decreasing its hydrophobicity. On the other hand, S93G is found on the β-strand leading into the CDR3 region. Most likely the removal of the hydrogen bond forming potential of serine leads to CDR3 loop dynamics that improve stability. Interestingly, out of the residues substituted only W103 interacted directly with the V_L domain.

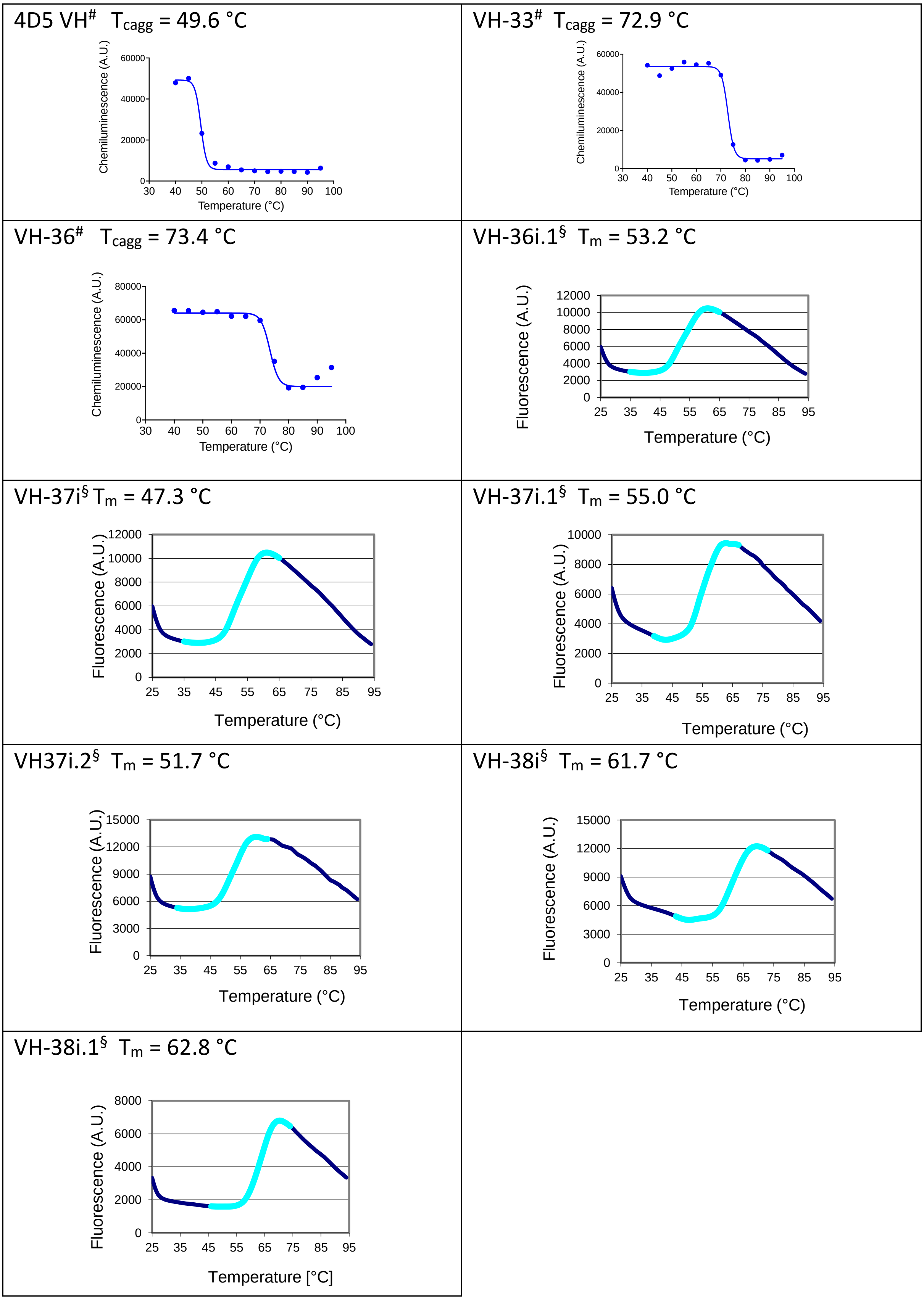

Supplementary Figure 3: Thermal stability curves for 4D5 and the CoFi and Hot-CoFi selected VH domain variants. Representative T_{CAGG} (#) and DSF T_m (§) stability curves obtained for the main 4D5 VH variants. For the DSF T_m curves, the fitted area is highlighted in cyan). A.U. = arbitrary units.

CDR1

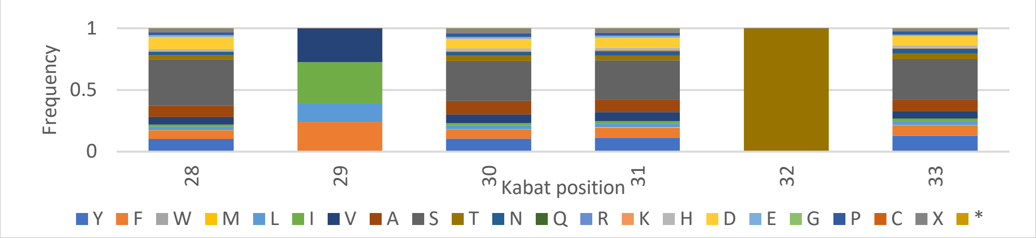

CDR2

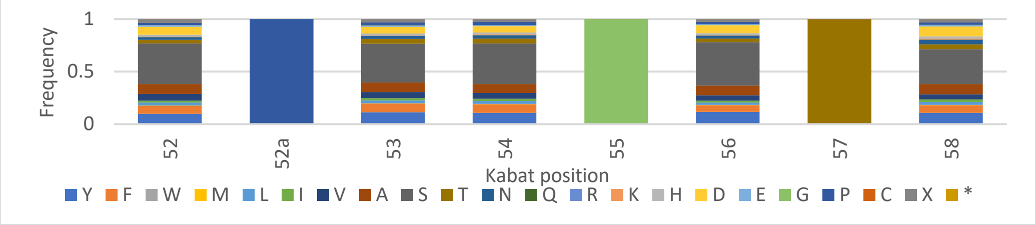

CDR3 (10 residues)

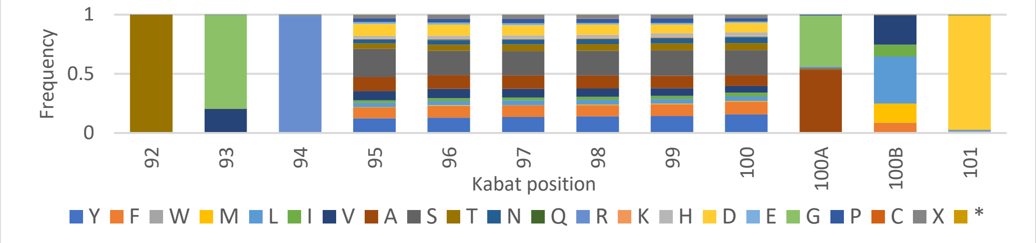

CDR3 (15 residues)

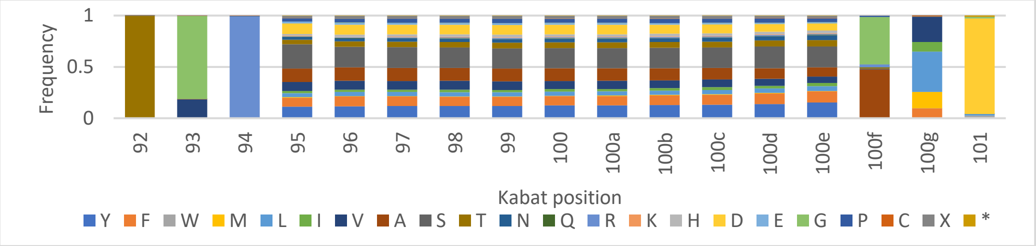

CDR3 (20 residues)

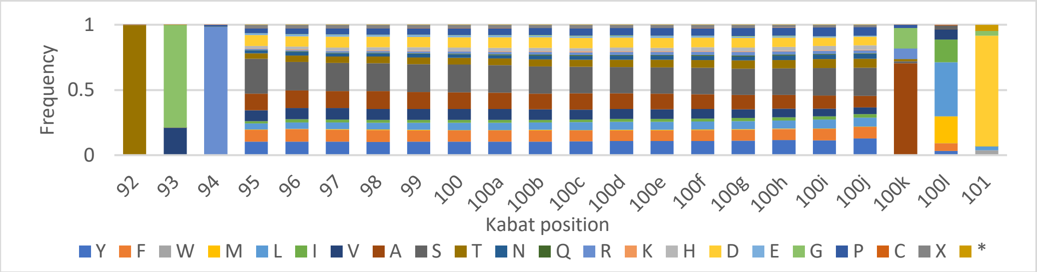

CDR3 (24 residues)

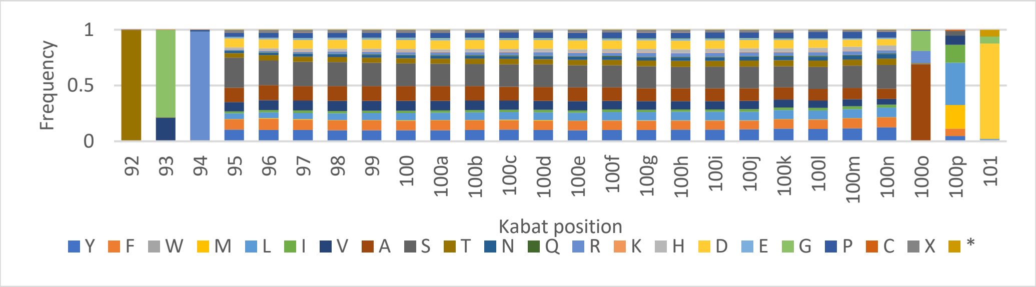

Supplementary Figure 4: Amino acid frequency analysis in each CDRs of the VH-DIF phage display library. 1,131,370 unique sequences were analyses and were obtained by next-generation sequencing**.** For CDR3, only analysis for four representative CDR lengths are shown. The amino acids are represented as single-letter codes, using the Kabat numbering scheme. X represents an amber codon (translated as E in the bacterial strain used) and * represents the stop codon.

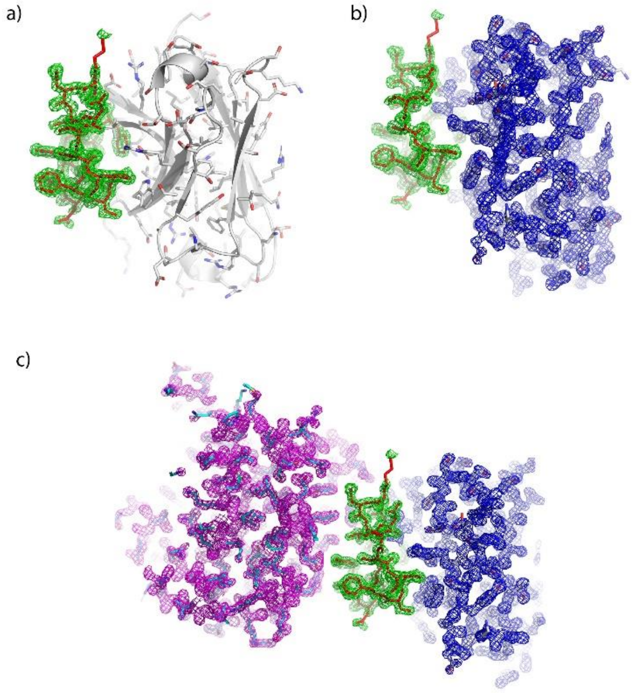

Supplementary Figure 5: **Complex structure of VH-1C5 with eIF4E.** Cartoon and stick presentation of the VH-1C5 structure from the complex with eIF4E (PDB ID: 7D6Y): **a)** The 2Fo-Fc electron density map contoured at 1.5σ for the CDR3 region that interacts with eIF4E is shown in green. **b)** 2Fo-Fc electron density map contoured at 1.5 σ of the main body of the VH domain highlighted in blue. **c)** Cartoon and stick presentation structure of VH-1C5 in complex with eIF4E. The magenta mesh highlights the 2Fo-Fc electron density map contoured at 1.5σ of eIF4E. The VH-1C5 domain is shown as illustrated in **a)** and **b)**.

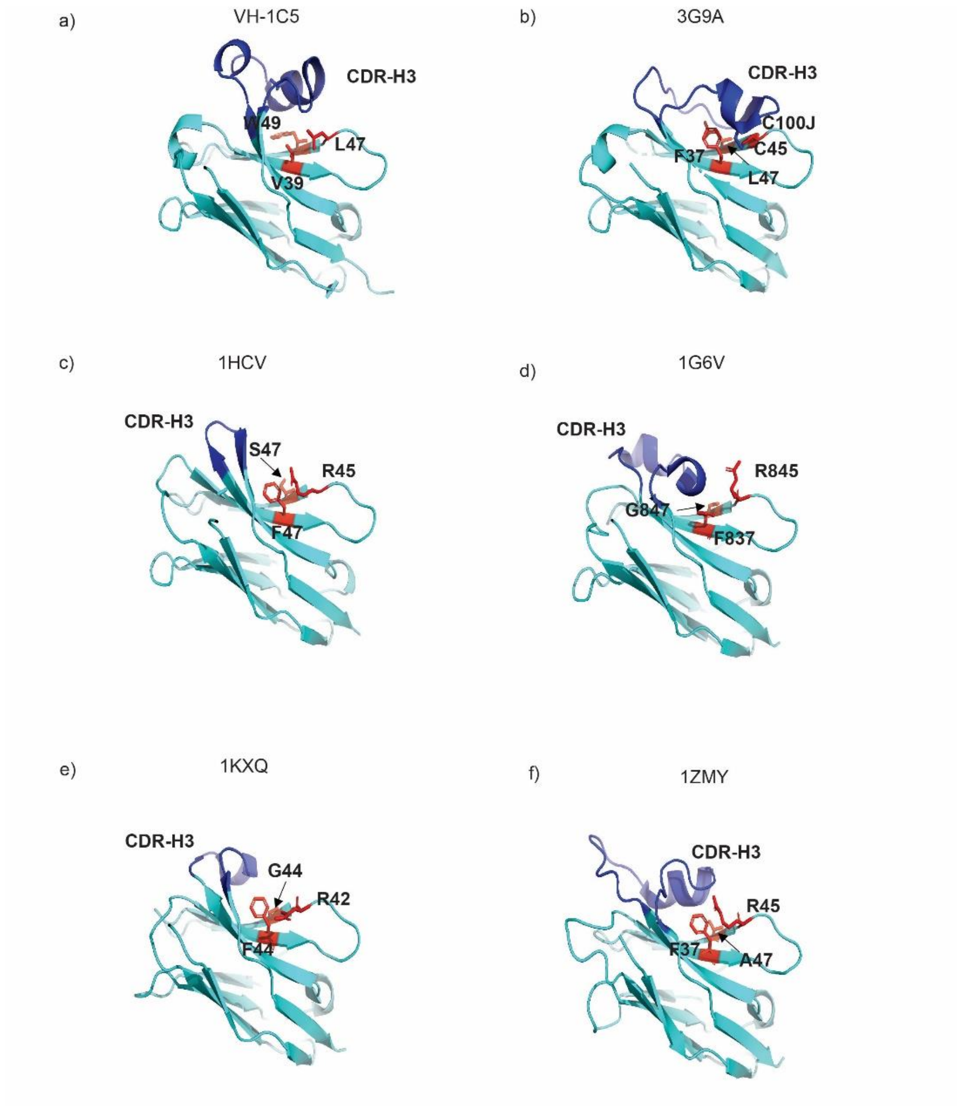

Supplementary Figure 6: Comparison of VH-1C5 CDR-H3 loop structure and packing interactions with several nanobodies: a) VH-1C5, B) eGFP interacting nanobody (PDB: 3G9A), c) α-subunit of human chorionic gonadotropin interacting nanobody (PDB: 1HCV), d) bovine carbonic anhydrase interacting nanobody (PDB: 1G6V), **e)** porcine pancreatic α-amylase interacting nanobody (PDB: 1KXQ) and f) hen egg white lysozyme interacting nanobody (PDB: 1ZMY) Nanobodies are variable domains derived from the heavy-chain only camelid antibodies that are monomeric and soluble. The structure of VH-1C5 is highly analogous to Nanobodies, where an extended CDR3 loop frequently folds back on to the protein to interact with and shield hydrophobic residues from the solvent. The CDR-H3 loop of VH-1C5 forms interactions with the residues V39, W49 and L37 that are located on the form VH:VL packing interface. These residues are highly conserved in VH domains to maintain VH-VL pairing, whilst in nanobodies these positions exhibit greater variability as they are not under this evolutionary constraint and therefore have greater potential to contribute to CDR-H3 loop packing interactions against the domain. For example, the conserved phenylalanine (highlighted in red) residue in b) to f) forms stabilising interactions with the CDR-H3 loop to help form the interaction interface. The other two residue positions (highlighted in red) show greater variation depending on the CDR-H3 loop size and its exposure to solvent. In the case of b) position 47 is a leucine and helps to stabilise the CDR3, whilst position 45 is a Cys and forms a disulphide bond with an opposing Cys in the CDR-H3 loop further rigidifying it. This contrast to c), where the presented CDR3 loop is much smaller, and the other positions are not involved in interactions with the loop and are much more solvent exposed. As a result, they (S47 and R45) are much more polar and play a greater role in solubilizing the monomeric domain.

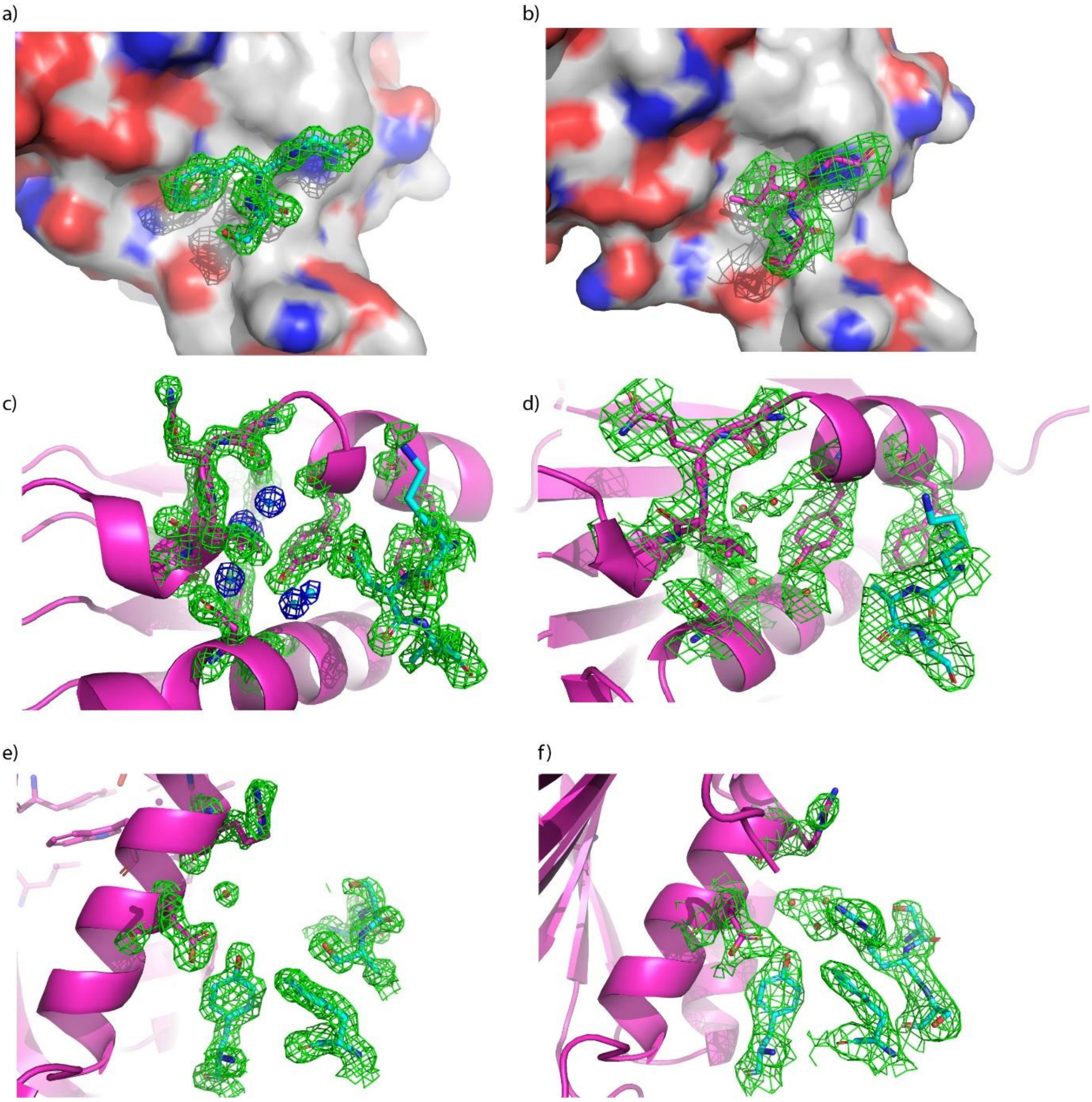

Supplementary Figure 7: Optimization of structural interaction of VH-S4 with eIF4E compared to VH-1C5. a) 2Fo-Fc electron density map (1.5σ) showing the F119 residue from VH-1C5 interacting with the eIF4E protein surface, and b) the corresponding 2Fo-Fc electron density map (1.1σ) of the I119 residue in VH-S4 interacting with the eIF4E protein surface. c) 2Fo-Fc electron density map (1.5σ) of the D103 residue from VH-1C5 and the network of eIF4E residues and water molecules it interacts with, compared to d) showing the corresponding A103 in VH-S4 and its interaction network. e) 2Fo-Fc electron density map of S107 from VH-1C5 and the local protein protein interface compared to f) the corresponding R107 in VH-S4, where the formation of a structured water network can be observed mediating interactions between the two proteins.

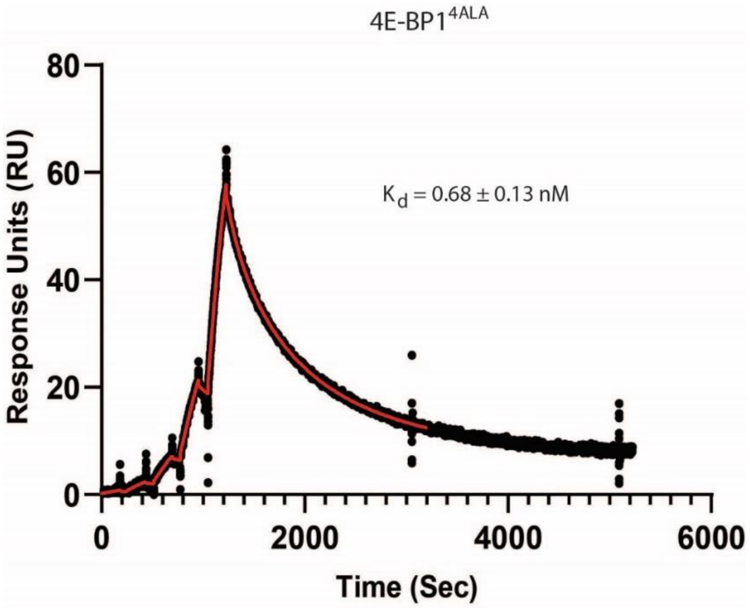

Supplementary Figure 8: 4E-BP1 binding interaction with eIF4E. Surface plasmon resonance sensogram of 4E-BP1^{4ALA} being titrated against eIF4E immobilised via amine coupling on a CM5 sensor chip (see materials and methods). Binding and kinetic parameters are shown in table 1. The 2-state binding curve fit is shown in red and was performed with BiaEvaluation (Cytiva, Ltd) software. K_d derived for the 2-state binding curve fit is denoted on graph. K_d value represents mean ± standard error with n = 3, independent experiments.

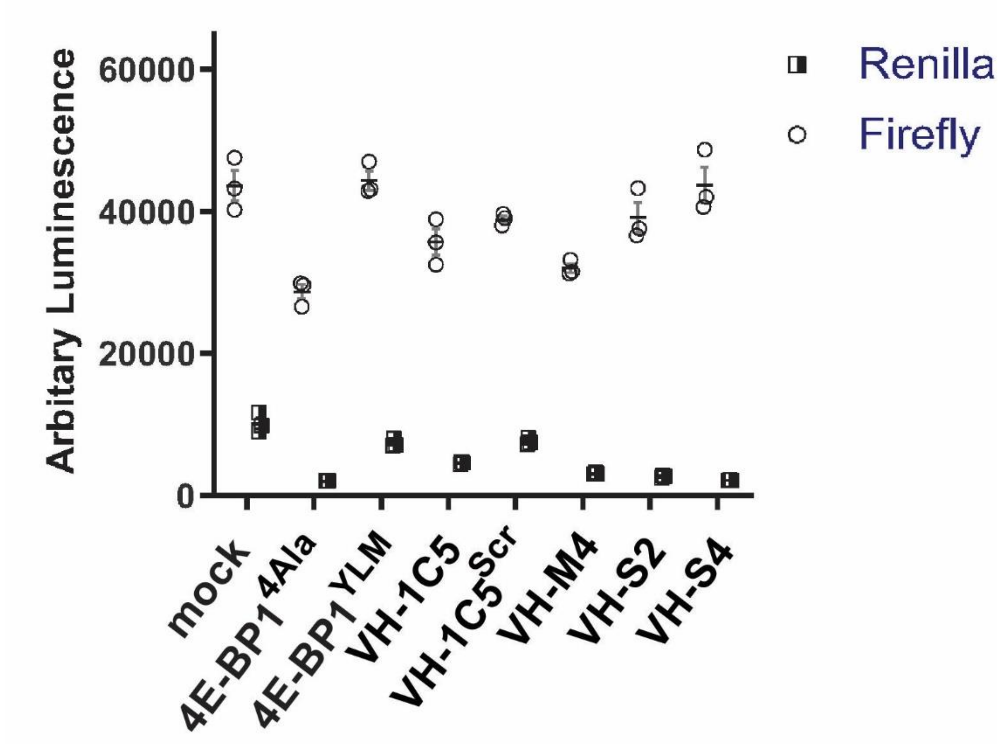

Supplementary Figure 9: eIF4E interacting VH domains disrupt cap-dependent translation in vitro. A bicistronic luciferase reporter, which measures the relative amount of cap-dependent translation (Renilla) to cap-independent translation (Firefly), was co-transfected with either empty vector (MOCK) or the denoted plasmids into HEK293 cells (see material and method). Renilla and Firefly luciferase activity was measured 48 h post transfection and plotted. These values plotted in the graph correspond to the ratio-metric values plotted in figure 5C in the main text. All values represent mean ± standard error with n = 3, independent experiments. Center line and limits denoted on the dot blot represent the mean value and standard deviation of the replicates, respectively. Source data is provided in the Source Data file.

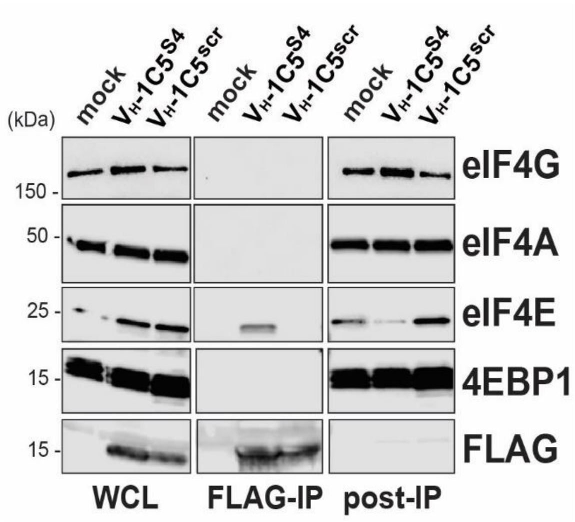

Supplementary Figure 10: VH-S4 is specific for eIF4E. Lysates were prepared from HEK293 cells and were either transfected with mock DNA, VH-S4 or VH-1C5^{Scr} and then used to perform anti-FLAG pull down followed by western blot analysis. eIF4G, eIF4A, eIF4E and 4E-BP1 antibodies were used to probe WCL (whole cell lysate), immune-precipitated protein (FLAG-IP) and cell lysate post-immunoprecipitation (post-IP) fractions. Protein levels of VH-S4 and VH-1C5^{Scr} were assessed with anti-FLAG. Western blot analysis was repeated twice to verify its reproducibility. Representative blot shown. Source data is provided in the Source Data file.

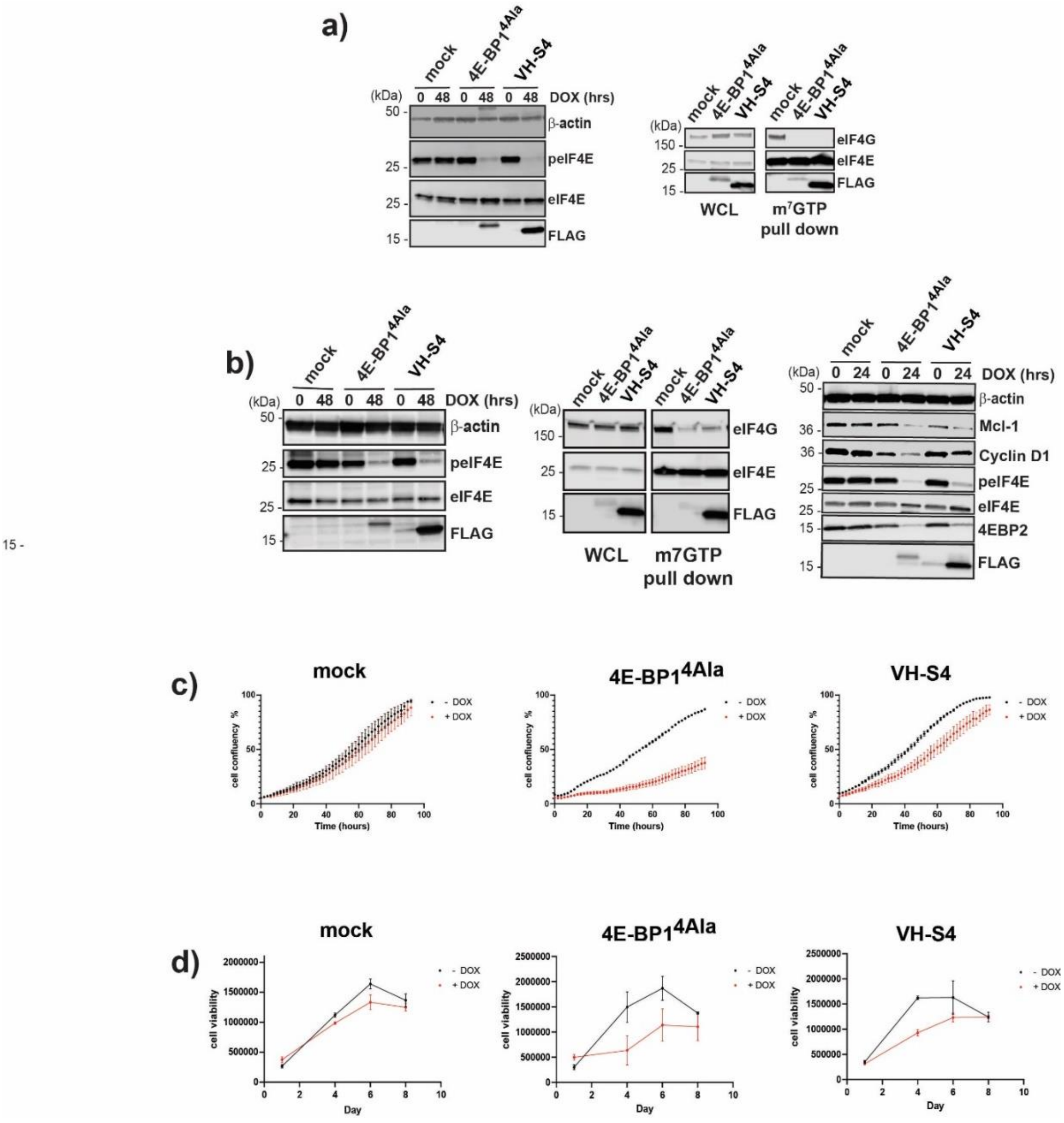

Supplementary Figure 11: Modulation of eIF4E mediated signaling pathways by VH-S4 leads to decreased cell proliferation. Stably transfected a) A375 cells and b) MBA-MD-231 cells harbouring doxycycline (DOX) inducible Mock, 4E-BP1^{4Ala} and VH-S4 were incubated for 48 hrs with or without [illegible]g/ml of doxycycline. Whole cell lysate (left-hand western blot) was blotted pre- and post- doxycycline induction and blotted for eIF4E, phosphorylated eIF4E, the FLAG-tagged 4E-BP1^{4Ala} and VH-S4 constructs and β-actin as a loading control. Parallel m^{7}GTP pull-down experiments (centre panel) were performed to analyse eIF4F complex formation cell lysate obtained from the DOX induced samples. In the right-hand side panel, MBA-MD-231 cell were treated with doxycycline and after 48 hours were assessed for the following protein levels, Mcl-1, Cyclin D1, peIF4E, eIF4E and 4E-BP2. B-actin was used as aloading control. c) MBA-MD-231 mock, 4E-BP1^{4Ala} and VH-S4 inducible cell line confluence was measured in presence or absence of doxycycline for the indicated time (hrs) using an Incucyte (EssenBiosciences). All experiments were repeated n = 3 times independently. Representative data shown d) A375 stable cell lines treated as in c) were assayed for viability over the indicated time (days). Experiments were repeated n =3 times, independently with representative time courses shown. All values on the plots represent mean ± SD (n=3, technical replicates). Experiments in c) and d) are representative and were performed n = 3 times. All western blot analyses were repeated twice to verify their reproducibility. Representative blots are shown. Source data for a, b, c and d is provided in the Source Data file.

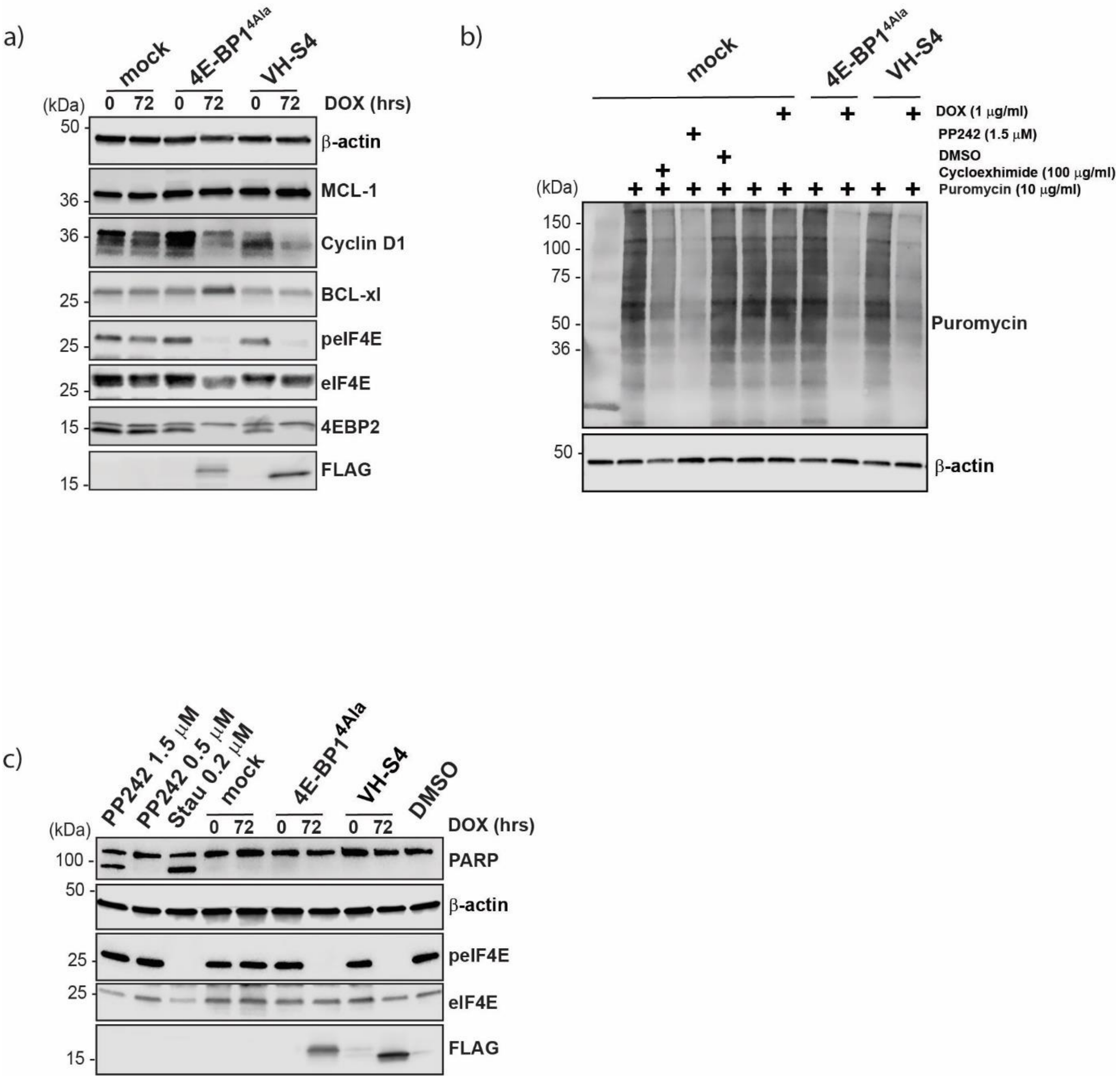

Supplementary Figure 12: eIF4F complex disruption leads to decrease in global protein synthesis in absence of apoptosis. a) Inducible A375 cells harbouring Mock, 4E-BP1^{4Ala} and VH-S4 constructs were incubated for 72 hrs with or without 1�g/ml of Doxycycline. Lysates were analysed by western blot using the anti-bodies indicated in the blot (for further details see materials and methods). Levels of 4E-BP1^{4Ala} and VH-S4 were assessed using anti-FLAG. b) Inducible A375 cells harbouring Mock, 4E-BP1^{4Ala} and VH-S4 constructs were treated with DOX, PP442, cycloheximide or vehicle control (DMSO 1% v/v) and then pulse labelled with puromycin post 24 hours after treatment. 20 ug of whole cell lysates were analysed for puoromycin labelling wing anti-Puoro. β-actin was blotted as a representative protein whose levels should not be affected by 4E-BP1^{4Ala} and VH-S4 induction. c) Inducible A375 cells harbouring Mock, 4E-BP1^{4Ala} and VH-S4 constructs were incubated for 72 hrs with or without 1�g/ml of Doxycycline, whilst mock cells were also treated with either PP242 or Staurosporine (Stau) with a residual DMSO concentration of 1 % (v/v). A DMSO vehicle control was also included. All western blot analyses were repeated twice to verify their reproducibility. Representative blots are shown and β-Actin was used as a loading control. Source data a, b and c is provided in the Source Data file.

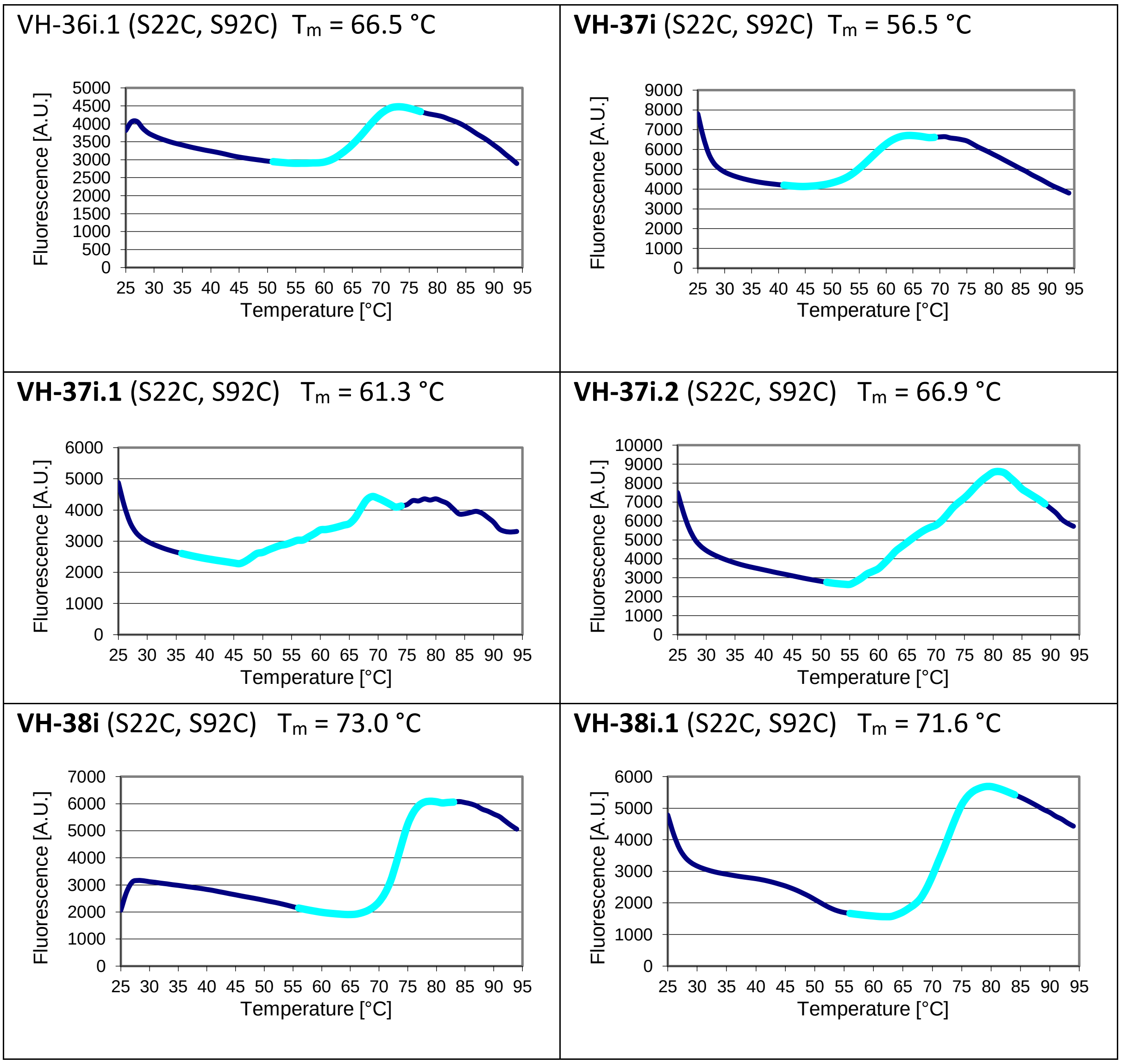

Supplementary Figure 13: Thermal stability curves for disulphide free VH domain variants with re-insertion of intra-domain disulphide bond. Representative DSF T_m stability curves are shown. T_m values were derived by curve fitting to the data points highlighted in cyan. VH domains with the re-insertion of the disulphide bond were approximately 10 °C more stable than their disulphide free counterparts in vitro (Supplementary Figure 3). A.U. = arbitrary units.

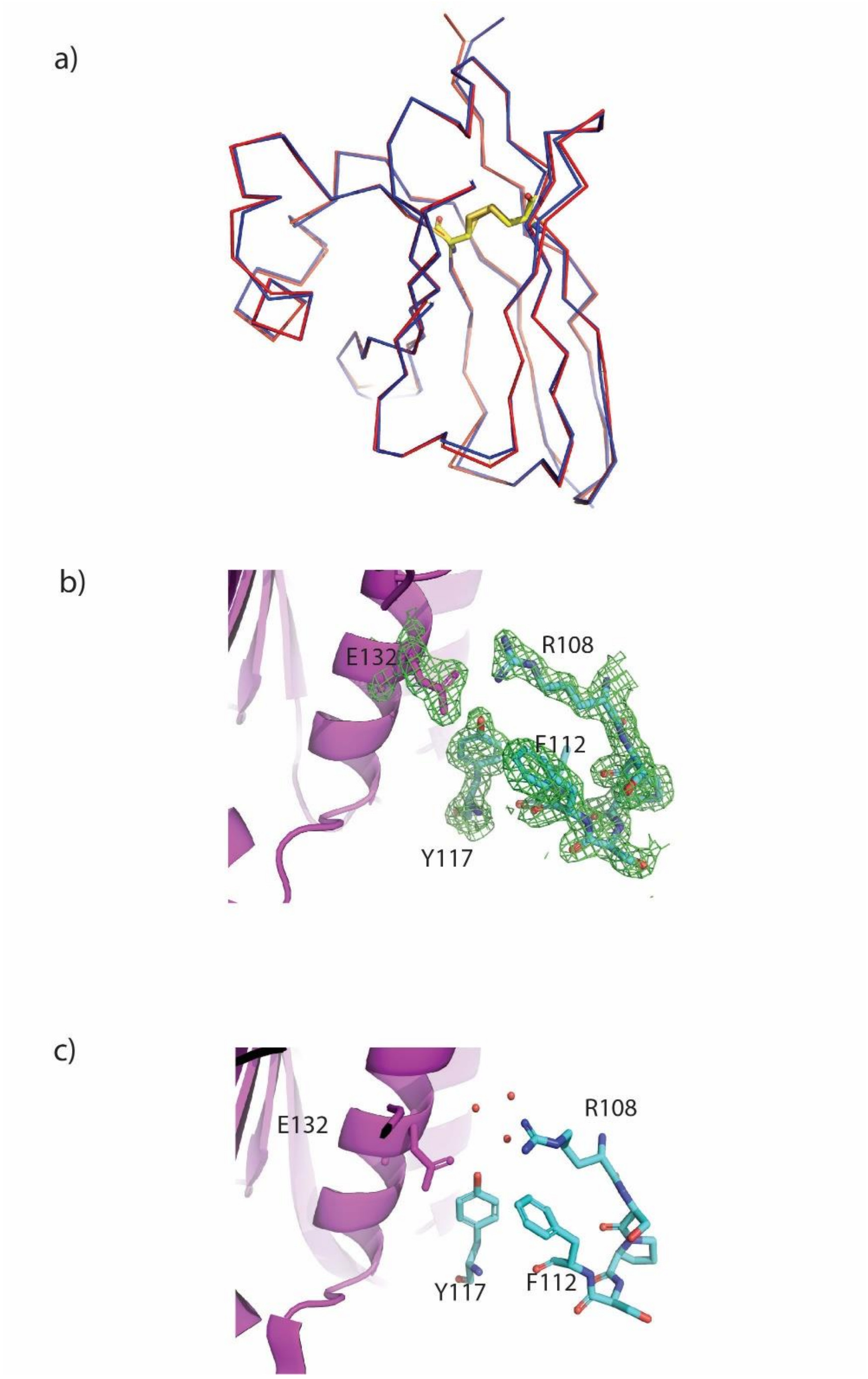

Supplementary Figure 14: Structural characterisation of VH-S4 bearing an intra-domain disulphide bond (VH-S4ss). a) Overlay of VH-S4ss (PDB ID: 7XTP, chain A) domain with the non-disulphide containing VH-S4 domain (PDB ID: 7D8B, chain B) demonstrating that the intra-disulphide bond causes no significant change to the overall fold of the autonomous VH domain (RMSD = 0.31 Å). VH-S4ss and VH-S4 are shown in blue and red, respectively, whilst the disulphide bond is highlighted in yellow. b) The S108R sidechain is found forming a direct electrostatic interaction with E132 and displacing the structured waters observed in the VH-S4 complex in c). In b) and c) eIF4E is shown in magenta, whilst the VH domain residues interacting with eIF4E are shown in cyan.

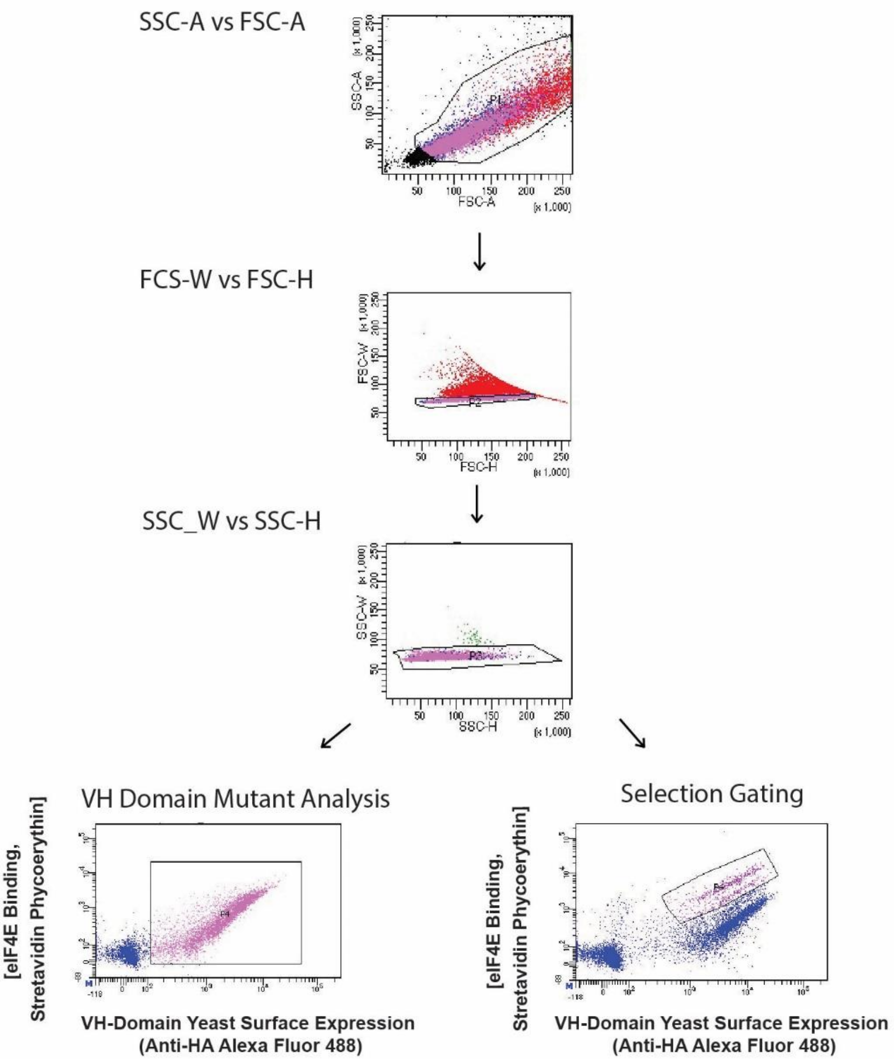

Supplementary Figure 15: Overview of FACs gating strategy for VH domain mutant analysis and affinity maturation selection. Yeast cells displaying VH domains on their surfaces were initially sorted via their FSC-A and SSC-A properties to select for intact cells. Next, the yeast population was sorted by the following 2 gates: 1) FSC-H and FSC-W, followed by 2) SSC-H and SSC-W to ensure non-aggregated cells were isolated. Finally, we used the desired fluorophore channels to gate and sort cells for further analysis. Yeast cells were incubated with anti-HA Ab Alexa Fluor 488 to measure VH domain expression and Streptavidin-phycoerythrin or neutravidin-phycoerythrin to detect biotinylated eIF4E molecules interacting with prospective VH domains on the yeast surface. For VH domain mutant binding analysis the mean fluorescence intensity was calculated from the highlighted box, whilst approximately the top 5% of the sorted yeast population positive for eIF4E binding was selected for expansion.

Supplementary Notes

PBB Evaluation Report (7D8B)

WORLDWIDE PDB PROTEIN DATA BANK

Full wwPDB X-ray Structure Validation Report ⓘ

CONFIDENTIAL VALIDATION REPORT

Oct 12, 2020 08:25 PM JST

PDB ID	:	7D8B
Title	:	Engineering Disulphide-Free Autonomous Antibody VH Domains to modulate intracellular pathways
Deposited on	:	2020-10-07
Resolution	:	2.46 Å(reported)

This is a Full wwPDB X-ray Structure Validation Report.

This report is produced by the wwPDB biocuration pipeline after annotation of the structure.

We welcome your comments at *validation@mail.wwpdb.org*
A user guide is available at
https://www.wwpdb.org/validation/2017/XrayValidationReportHelp
with specific help available everywhere you see the ⓘ symbol.

The following versions of software and data (see references ⓘ) were used in the production of this report:

MolProbity	:	4.02b-467
Xtriage (Phenix)	:	1.13
EDS	:	2.14.6
Percentile statistics	:	20191225.v01 (using entries in the PDB archive December 25th 2019)
Refmac	:	5.8.0158
CCP4	:	7.0.044 (Gargrove)
Ideal geometry (proteins)	:	Engh & Huber (2001)
Ideal geometry (DNA, RNA)	:	Parkinson et al. (1996)
Validation Pipeline (wwPDB-VP)	:	2.14.6

PBB Evaluation Report (7D6Y)

WORLDWIDE PDB PROTEIN DATA BANK

Full wwPDB X-ray Structure Validation Report ⓘ

CONFIDENTIAL VALIDATION REPORT

Oct 13, 2020 01:13 PM JST

PDB ID	:	7D6Y
Title	:	eIF4E in Complex with a Disulphide-Free Autonomous VH Domain
Deposited on	:	2020-10-02
Resolution	:	1.67 Å(reported)

This is a Full wwPDB X-ray Structure Validation Report.

This report is produced by the wwPDB biocuration pipeline after annotation of the structure.

We welcome your comments at *validation@mail.wwpdb.org*
A user guide is available at
https://www.wwpdb.org/validation/2017/XrayValidationReportHelp
with specific help available everywhere you see the ⓘ symbol.

The following versions of software and data (see references ⓘ) were used in the production of this report:

MolProbity	:	4.02b-467
Mogul	:	1.8.5 (274361), CSD as541be (2020)
Xtriage (Phenix)	:	1.13
EDS	:	2.14.6
buster-report	:	1.1.7 (2018)
Percentile statistics	:	20191225.v01 (using entries in the PDB archive December 25th 2019)
Refmac	:	5.8.0158
CCP4	:	7.0.044 (Gargrove)
Ideal geometry (proteins)	:	Engh & Huber (2001)
Ideal geometry (DNA, RNA)	:	Parkinson et al. (1996)
Validation Pipeline (wwPDB-VP)	:	2.14.6

PBB Evaluation Report (7XTP)

Full wwPDB X-ray Structure Validation Report (i)

May 26, 2022 – 01:43 PM JST

PDB ID	:	7XTP
Title	:	eIF4E in Complex with a Disulphide-Free Autonomous VH Domain
Deposited on	:	2022-05-17
Resolution	:	1.83 Å(reported)

This wwPDB validation report is for manuscript review

This is a Full wwPDB X-ray Structure Validation Report.

This report is produced by the wwPDB biocuration pipeline after annotation of the structure.

We welcome your comments at *validation@mail.wwpdb.org*
A user guide is available at
https://www.wwpdb.org/validation/2017/XrayValidationReportHelp
with specific help available everywhere you see the (i) symbol.

The following versions of software and data (see references (i)) were used in the production of this report:

MolProbity	:	4.02b-467
Mogul	:	1.8.5 (274361), CSD as541be (2020)
Xtriage (Phenix)	:	1.13
EDS	:	2.28.1
buster-report	:	1.1.7 (2018)
Percentile statistics	:	20191225.v01 (using entries in the PDB archive December 25th 2019)
Refmac	:	5.8.0158
CCP4	:	7.0.044 (Gargrove)
Ideal geometry (proteins)	:	Engh & Huber (2001)
Ideal geometry (DNA, RNA)	:	Parkinson et al. (1996)
Validation Pipeline (wwPDB-VP)	:	2.28.1

For Manuscript Review
