# Supplementary material for: Engineering an autonomous VH domain to modulate intracellular pathways and to interrogate the eIF4F complex
Source: Nat Commun. 2022 Aug 18;13:4854. doi: 10.1038/s41467-022-32463-1 (PMC9388512; doi:10.1038/s41467-022-32463-1)
Supplement: Supplementary file 2 — Reporting Summary [file 41467_2022_32463_MOESM2_ESM.pdf]

nature portfolio

Corresponding author(s): Christopher J Brown

Last updated by author(s): Jul 1, 2022

Reporting Summary

Nature Portfolio wishes to improve the reproducibility of the work that we publish. This form provides structure for consistency and transparency in reporting. For further information on Nature Portfolio policies, see our Editorial Policies and the Editorial Policy Checklist.

Statistics

For all statistical analyses, confirm that the following items are present in the figure legend, table legend, main text, or Methods section.

n/a	Confirmed	
[ ]	[x]	The exact sample size (*n*) for each experimental group/condition, given as a discrete number and unit of measurement
[ ]	[x]	A statement on whether measurements were taken from distinct samples or whether the same sample was measured repeatedly
[x]	[ ]	The statistical test(s) used AND whether they are one- or two-sided<br>*Only common tests should be described solely by name; describe more complex techniques in the Methods section.*
[x]	[ ]	A description of all covariates tested
[x]	[ ]	A description of any assumptions or corrections, such as tests of normality and adjustment for multiple comparisons
[x]	[ ]	A full description of the statistical parameters including central tendency (e.g. means) or other basic estimates (e.g. regression coefficient) AND variation (e.g. standard deviation) or associated estimates of uncertainty (e.g. confidence intervals)
[x]	[ ]	For null hypothesis testing, the test statistic (e.g. *F*, *t*, *r*) with confidence intervals, effect sizes, degrees of freedom and *P* value noted<br>*Give P values as exact values whenever suitable.*
[x]	[ ]	For Bayesian analysis, information on the choice of priors and Markov chain Monte Carlo settings
[x]	[ ]	For hierarchical and complex designs, identification of the appropriate level for tests and full reporting of outcomes
[x]	[ ]	Estimates of effect sizes (e.g. Cohen's *d*, Pearson's *r*), indicating how they were calculated

Our web collection on statistics for biologists contains articles on many of the points above.

Software and code

Policy information about availability of computer code

Data collection	BD FACSDiva6.1, XDS Version Novenber 11, 2017,
Data analysis	PYMOL v2.5.2, AMBER14, Graphpad Prism 8.0, Wincoot (version 8.9), CCP4 (version 7), Phaser 2.7, REFMAC (version 5), ImageJ v1.53, Biacore T100 Evaluation software v2.0.4., Procheck v3.5, Blitz Pro software 1.3.0.5

For manuscripts utilizing custom algorithms or software that are central to the research but not yet described in published literature, software must be made available to editors and reviewers. We strongly encourage code deposition in a community repository (e.g. GitHub). See the Nature Portfolio guidelines for submitting code & software for further information.

Data

Policy information about availability of data

All manuscripts must include a data availability statement. This statement should provide the following information, where applicable:

- Accession codes, unique identifiers, or web links for publicly available datasets
- A description of any restrictions on data availability
- For clinical datasets or third party data, please ensure that the statement adheres to our policy

RReagents used in this manuscript are available from the corresponding authors upon reasonable request. Structure factors and coordinates have been deposited in the Protein Data Bank under accession codes 7D88, 7D6Y and 7XTP for the eIF4E:VH-1C5, eIF4E VH-S4 and eIF4E:VHS4ss complexes, respectively. The source data generated in this study are provided in the Supplementary information or Source Data file. Other data is available upon reasonable request

Human research participants

Policy information about studies involving human research participants and Sex and Gender in Research.

Reporting on sex and gender	N/A
Population characteristics	N/A
Recruitment	N/A
Ethics oversight	N/A

Note that full information on the approval of the study protocol must also be provided in the manuscript.

Field-specific reporting

Please select the one below that is the best fit for your research. If you are not sure, read the appropriate sections before making your selection.

- [x] Life sciences
- [ ] Behavioural & social sciences
- [ ] Ecological, evolutionary & environmental sciences

For a reference copy of the document with all sections, see nature.com/documents/nr-reporting-summary-flat.pdf

Life sciences study design

All studies must disclose on these points even when the disclosure is negative.

Sample size	No statistical method was used to pre-determine sample size, but sample size is similar to sample sizes routinely used in the field.
Data exclusions	No data exclusion were used.
Replication	Number of repeats for each experiment are stated in figure legends. All repetition reproduced previous results.
Randomization	No randomization was used.
Blinding	The investigators were not blinded to allocation during experiments.

Reporting for specific materials, systems and methods

We require information from authors about some types of materials, experimental systems and methods used in many studies. Here, indicate whether each material, system or method listed is relevant to your study. If you are not sure if a list item applies to your research, read the appropriate section before selecting a response.

Materials & experimental systems

n/a	Involved in the study	
☐	☒	Antibodies
☐	☒	Eukaryotic cell lines
☒	☐	Palaeontology and archaeology
☒	☐	Animals and other organisms
☒	☐	Clinical data
☒	☐	Dual use research of concern

Methods

n/a	Involved in the study	
☒	☐	ChIP-seq
☐	☒	Flow cytometry
☒	☐	MRI-based neuroimaging

Antibodies

Antibodies used

1) Anti-eIF4E (phospho S209), Cat #: AB76256, clone EP2151Y, Abcam, Dilution used 1:5000.
2) Anti phospho 4EBP1 threonine 36/46, Cat #: 2855, clone 236B4, Cell signaling Technology , Dilution used 1:1000.
3) Anti p44/42 MAPK, Cat #: 9102, clone number not provided, Cell signaling Technology , Dilution used 1:1000.
4) Anti pAKT ser473, Cat #: 9271, clone number not provided Cell signaling Technology 1:1000.
5) Anti eIF4E, Cat #: 2067, Clone C46H6, Cell signaling Technology, Dilution used 1:500.
6) Anti 4EBP1, Cat #: 9644, Clone 53H11, Cell signaling Technology, Dilution used 1:4000.
7) Anti phospho p44/42 Cat #: 9101, clone number not provided, Cell signaling Technology, Dilution used 1:1000.

8) Anti AKT, Cat #: 9272, clone number not provided, Cell signaling Technology, Dilution used 1:1000.
9) Anti eiF4G1, Cat #: 2858, clone number not provided, Cell signaling Technology, Dilution used 1:500.
10) Anti cyclin D1, Cat #: 2988, clone 92G2, Cell signaling Technology, Dilution used 1:500.
11) Anti Mcl-1 , Cat #: 5453, clone D35A5, Cell signaling Technology, Dilution used 1:1000.
12) Anti 4EBP2, Cat #: 2845, clone number not provided, Cell signaling Technology, Dilution used 1:1000.
13) Anti Bcl-Xl , Cat #: 2764, clone number 54H6, Cell signaling Technology, Dilution used 1:1000.
14) Anti Parp, Cat #: 9532, clone 46D11, Cell signaling Technology, Dilution used 1:1000.
15) Anti Stat-1, Cat #: 9172, clone number not provided, Cell signaling Technology, Dilution used 1:1000.
16) Anti β-actin peroxidase, Cat #: A3854, clone AC-15, Sigma Aldrich, Dilution used 1:10000.
17) Anti-FLAG peroxidase , Cat #: A8592, clone M2, Sigma Aldrich, Dilution used 1: 1000
18) Anti Phospho-S6 Ribosomal subunit Ser235/236, Cat #: 2211, clone number not provided, Cell signaling Technology, Dilution used 1: 1000
19) Anti S6 Ribosomal subunit, Cat #: 2317, clone 54D2, Cell signaling Technology, Dilution used 1: 1000.
20) anti-M13 antibody HRP conjugate, Cat #: 27-9421-01, no clone number provided, Cytiva, Dilution used 1/7000.
21) Anti-HA Ab Alexa Fluor 488, , Cat #: A-11039, ThermoFisher Scientific, clone number not provided, Dilution used 1:200)

Validation

1) validation by Western blot using cell lysate obtained from cells treated with phosphorylation inhibitors for POI (Frosi et al 2019, Xu M et al 2020; Megat S et al 2019)
2) validation by Western blot using cell lysate obtained from cells treated with phosphorylation inhibitors for POI(Grigore et al., 2020; Andrews G et al., 2020)
3) validation in human cell lysates by Western blot (Daniel-Garcia et al., 2020; Bele et al., 2020;)
4) validation by Western blot using cell lysate obtained from cells treated with phosphorylation inhibitors for POI (Sook Ann et al. 2021; Ritter et al., 2020)
5) validation in human cell lysates by Western blot (Ham et al 2020; Pringle et al., 2019)
6)validation in human cell lysates by Western blot (Smith et al 2021; Meena et al., 2020)
7)validation by Western blot using cell lysate obtained from cells treated with phosphorylation inhibitors for POI(Ognibene et al., 2020; Brown et al., 2020)
8) validation by Western blot using cell lysate obatined from cells treated with either non-targeted (-) or SignalSilence® Akt siRNA I (+). Statement from manufacturer's website: Akt Antibody detects endogenous levels of total Akt1, Akt2 and Akt3 proteins. The antibody does not cross-react with related kinases. Validated in western blots analysis (Xin, Shan et al. “MS4A15 drives ferroptosis resistance through calcium-restricted lipid remodeling.” Cell death and differentiation vol. 29,3 (2022): 670-686.).
9) validation in human cell lysates by Western blot (Pringle et al., 2019; Gallagher et al., 2019)
10) validation in human cell lysates by Western blot (Yang et al. 2020; Sun et al., 2020)
11) validation in human cell lysates by Western blot (Gao et al. 2020; Yoon et al., 2020)
12) validation in human cell lysates by Western blot (Wiebe et al. 2019; Iezaki et al., 2018)
13) validation in human cell lysates by Western blot (Balakrishnan et al., 2020; Panagaki et al., 2020)
14) validation in human cell lysates by Western blot using PARP knockout cell line extracts (Zhao et al 2021; You et al., 2020)
15) validation in human cell lysates by Western blot using STAT-1 knockout cell line extracts (Xu et al., 2020)
16)validation in human cell lysates by Western blot (Eschenburg et al., 2012)
17)validation in human cell lysates by Western blot (Waterfield et al 2014; Chaumet et al., 2015)
18)validation by Western blot using cell lysate obtained from cells treated FBS to modulate POI phosphorylation (Sun et al., 2020)
19) validation in human cell lysates by Western blot (Sevigny et al., 2020; Kumar et al., 2020)
20) validation in ELISA (Huo, J., Mikolajek, H., Le Bas, A. et al. A potent SARS-CoV-2 neutralising nanobody shows therapeutic efficacy in the Syrian golden hamster model of COVID-19. Nat Commun 12, 5469 (2021).)
21) Validation in yeast surface display experiments (Chao, G. et al. Isolating and engineering human antibodies using yeast surface display. Nat. Protoc. 1, 755–768 (2006).

Eukaryotic cell lines

Policy information about cell lines and Sex and Gender in Research

Cell line source(s)	293FT (thermo Fisher Scientific); A375 (ATCC); MDA MB231 (ATCC); Hela (ATCC)
Authentication	All cell lines were purchased from the indicated company provider and were certified as being authentic. All cell lines were not internally authenticated.
Mycoplasma contamination	All cell lines were mycoplasma free. Tested weekly.
Commonly misidentified lines (See ICLAC register)	*Name any commonly misidentified cell lines used in the study and provide a rationale for their use.*

Flow Cytometry

Plots

Confirm that:

- [x] The axis labels state the marker and fluorochrome used (e.g. CD4-FITC).
- [x] The axis scales are clearly visible. Include numbers along axes only for bottom left plot of group (a 'group' is an analysis of identical markers).
- [x] All plots are contour plots with outliers or pseudocolor plots.
- [x] A numerical value for number of cells or percentage (with statistics) is provided.

Methodology

Sample preparation	EBY100 yeast strain was purchased from a commercial vendor. Yeast were washed and incubated with 200nM of biotinylated eIF4E. Yeast were then washed and resuspended with PBSA (phosphate-buffered saline with bovine serum albumin) 2 µM of unbiotinylated eIF4E (to prevent further association of labelled target) and incubated at room temperature for 8 minutes to enable dissociation of biotinylated eIF4E. Cells were washed in PBSA, resuspended in PBSA with Anti-HA Ab Alexa Fluor 488 (Invitrogen) and Streptavidin-phycoerythrin or neutravidin-phycoerythrin (ThermoFisher Scientific) for 10 min, and incubated on ice. Labelled cells were washed with 1 mL PBSA, resuspended in 0.5–2.0 mL PBSA and analysed by flow cytometry using an Aria (Becton Dickinson) cytometer. Cells positive for anti-HA and eIF4E were selected and sorted.
Instrument	BD FACSAria
Software	BD FACSDiva6.1
Cell population abundance	At the final stage of sorting approximately 40% of the initial sample of yeast cells was sampled.
Gating strategy	The first gate was used to sort cells via their FSC-A and SSC-A properties. This selected for intact cells and usually isolated approx. 60% of the yeast population under analysis/selection. Next, the yeast population was sorted by the following 2 gates: 1) FSC-H and FSC-W, followed by 2) SSC-H/SSC-W to ensure non-aggregated cells were isolated. By this point in the gating strategy approx. 40% of the starting sample remained. Finally, we used the desired fluorophore channels to gate and sort cells for further analysis.

- [x] Tick this box to confirm that a figure exemplifying the gating strategy is provided in the Supplementary Information.
